# QT Interval Variability Index and QT Interval Duration in Different Sleep Stages: Analysis of Polysomnographic Recordings in Nonapneic Male Patients

**DOI:** 10.1155/2015/963028

**Published:** 2015-11-29

**Authors:** Moonika Viigimae, Deniss Karai, Peeter Pirn, Kristjan Pilt, Kalju Meigas, Jyri Kaik

**Affiliations:** ^1^Technomedicum, Tallinn University of Technology, Ehitajate tee 5, 19086 Tallinn, Estonia; ^2^Tallinn Health Care College, Kännu 67, 13418 Tallinn, Estonia; ^3^Mae Pindmaa Sleep Clinic, Narva mnt 112, 10127 Tallinn, Estonia

## Abstract

The aim of the study was to determine whether different sleep stages, especially REM sleep, affect QT interval duration and variability in male patients without obstructive sleep apnea (OSA). Polysomnographic recordings of 30 patients were analyzed. Beat-to-beat QT interval variability was calculated using QTV index (QTVI) formula. For QTc interval calculation, in addition to Bazett's formula, linear and parabolic heart rate correction formulas with two separate *α* values were used. QTVI and QTc values were calculated as means of 2 awake, 3 NREM, and 3 REM sleep episodes; the duration of each episode was 300 sec. Mean QTVI values were not statistically different between sleep stages. Therefore, elevated QTVI values found in patients with OSA cannot be interpreted as physiological sympathetic impact during REM sleep and should be considered as a risk factor for potentially life-threatening ventricular arrhythmias. The absence of difference of the mean QTc interval values between NREM and REM stages seems to confirm our conclusion that sympathetic surges during REM stage do not induce repolarization variability. In patients without notable structural and electrical remodeling of myocardium, physiological elevation in sympathetic activity during REM sleep remains subthreshold concerning clinically significant increase of myocardial electrical instability.

## 1. Introduction

Decline in sleep quality is a generally accepted modulator of cardiovascular function. During the last decades, there is a growing interest in certain breathing-related sleep disorders, especially obstructive sleep apnea (OSA), due to their established association with major cardiovascular diseases, cardiac arrhythmias, and sudden cardiac death (SCD) [1–4]. In 2005, Gami et al. [5] were the first to emphasize that, in contrast to the general population, the patients with OSA have a peak in sudden cardiac death occurrence at night during sleeping hours, suggesting the involvement of sleep related mechanisms.

Although the exact pathophysiological factors linking OSA and cardiovascular risk are not completely identified so far, several evidences have revealed that chronic sleep fragmentation and intermittent hypoxia may cause a shift in the sympathovagal balance toward a sympathetic predominance and a vagal withdrawal [6], giving rise to increased myocardial electrical instability (MEI).

Myocardial electrical instability, that is, a predisposition to potentially life-threatening ventricular arrhythmias, can be indirectly assessed by numerous noninvasive parameters, including those reflecting ventricular repolarization prolongation and inhomogeneity. Different QT interval dispersion, prolongation, and variability estimation models used in clinical practice have demonstrated their utility for high-risk patient identification over the last two decades [7–9]. Several mathematical formulas have been proposed to describe the physiological pattern of the QT/RR relation and QT interval heart rate adaptation. Despite the fact that its limitations are often pointed out, Bazett's formula from 1920 is the one used most frequently.

Malik and coauthors [10] have converted several QT/RR regression models (linear, hyperbolic, parabolic, logarithmic, shifted logarithmic, and exponential models) to generic heart rate correction formulas to provide QTc interval values that are independent of the corresponding RR interval values. This approach seems to have created a predisposition for individually optimised heart rate correction.

Beat-to-beat QT interval variability is most often measured using QT variability index (QTVI), proposed by Berger et al. in 1997 [7]. The index quantifies the magnitude of QT interval fluctuations, normalized by both the mean QT duration and the magnitude of heart rate fluctuations. This index has been utilized as a predictor of malignant ventricular arrhythmias in various cardiac and noncardiac conditions, such as congestive heart failure [11], coronary artery disease [12], and panic disorders [13]. Atiga et al. [14] were the first to demonstrate the association between increased level of QT variability and arrhythmic events. In their study, the QTVI was the only clinical variable that identified patients with a previous incidence of SCD in multivariate regression model, outperforming as predictor of several well-known parameters, such as QT interval spatial dispersion, T-wave alternans, ventricular tachycardia inducibility, signal-averaged ECG, heart rate variability, and left ventricular ejection fraction.

Although ventricular repolarization determination methods have been recognized as powerful predictors of SCD in heart disease population, only few studies have examined QT interval properties in OSA patients. In these studies, the results demonstrated QT interval prolongation and increased inhomogeneity [15, 16]. Kilicaslan et al. [17] reported prolongation of a certain QT interval fragment, T-wave peak-to-end, in patients with OSA. Baumert et al. [16] demonstrated that QTVI values, calculated from polysomnographic (PSG) recordings, were associated with sleep apnea severity, but not with sleep stages.

To the best of our knowledge, very limited data [18] is available concerning QT interval duration and variability changes in patients without OSA during various sleep stages. Since rapid eye movement (REM) sleep is characterized by sympathetic surges and elevated sympathetic tone influences both the QT interval duration [19] and QT interval variability [20, 21], the assessment of modulatory effects of various sleep stages on ventricular repolarization parameters per se may add more understanding to arrhythmia genesis in OSA patients.

The novelty of our study lies in applying various QT interval correction formulas and QT variability index while awake and during nonrapid eye movement (NREM) as well as REM sleep stages in nonapneic patients.

The aim of the study was to determine whether different sleep stages, especially REM sleep, affect QT interval duration and variability in male patients without OSA.

## 2. Methods

### 2.1. Study Population

The study population included 30 male patients aged between 21 and 60 years who had undergone diagnostic overnight polysomnography for suspected OSA in 2013 and 2014. Patients were selected on the basis of stored PSG recordings and clinical characteristics. The inclusion criteria were as follows: male gender, ECG revealing sinus rhythm, absence of OSA (apnea-hypopnea index < 5), and clinically significant comorbidities, without excessive obesity (defined as BMI ≥ 35 kg/m^2^). A few patients with treatment-controlled arterial hypertension were included in the study. None of the patients was receiving I and III class antiarrhythmic medications known to affect the QT interval and other drugs that could potentially prolong ventricular repolarization (e.g., antihistaminic, psychotropic, or antibiotic medications). In this study, we did not measure the sleep quality but focused on QT interval within certain sleep stages. To the best of our knowledge, commonly used hypotensive drugs such as angiotensin-converting enzyme inhibitors, angiotensin II receptor blockers, and beta blockers have not demonstrated significant impact on neither QT interval nor sleep stages.

The study was approved by the Tallinn Medical Research Ethics Committee at the National Institute for Health Development.

### 2.2. Polysomnographic Recordings Analysis and Signal Preprocessing

The PSG data was obtained from routine diagnostic procedures performed at the Mae Pindmaa Sleep Clinic (Tallinn, Estonia). The signals were registered with polysomnography recorder Rembrandt Monet Artist SLP EZ 24 (Medicare Automation B.V., Netherlands). In all patients the following signals were recorded simultaneously: electrooculography (EOG), electrocardiography (ECG), electroencephalography (EEG), submental and anterior tibialis electromyography (EMG), oxygen saturation (SpO_2_), oronasal airflow, thoracic and abdominal respiration movements, snoring, and video monitoring. Sampling frequency for physiological signals was 200 Hz. Electrodes and sensors were placed by a professional polysomnography nurse. The duration of the recordings was from 8 to 9 hours. All PSG recordings were analyzed by a well-trained, certified, and experienced sleep technician. Data processing was performed using the Rembrandt Analysis Manager (version 7.5, Medicare Automation B.V., Netherlands). Sleep stages were confirmed manually using standardized procedures in accordance with the technical report of American Academy of Sleep Medicine published in 2007 [22]. Recording segments presenting frequent artefacts were excluded from the analysis. In each recording, two awake, three REM, and three NREM (stage II) sleep episodes (300 seconds each) suitable for processing were selected.

### 2.3. ECG Recording Analysis

QT interval duration and variability assessment in different sleep stages was performed at polysomnographic ECG lead II. ECG recordings were anonymized and converted to EDF format. Signals were resampled to sampling frequency of 256 Hz due to the EDF format converter provided by producer of PSG recorder. Recorded signals were preprocessed in LabVIEW (National Instruments, USA) environment, applying signal converting Physionet toolkit [23]. R-peaks were detected and extrasystoles identified using Pan-Tompkins algorithm implemented by P. S. Hamilton (Eplimited Ltd., USA). RR intervals were converted to Normal-to-Normal (NN) intervals. Ectopic beats, as well as pre- and postextrasystolic beats, were excluded from the analysis. T-wave location and type were detected using Ecgpuwave software [24]. As a measure of heart rate variability we computed the standard deviation of normal RR intervals.

We determined mean QT interval duration and T-wave apex to T-wave end interval (Tp-e) duration in all episodes. Only monophasic well-defined T-waves were accepted for measurement. T wave apex and T wave end points' detection were visually verified by an experienced investigator. The end of T wave was determined using the downslope tangent method described previously [25].

QT variability index was evaluated by Berger's formula [7], which is calculated for each subject as the logarithm of the ratio of normalized QT variance to heart rate variance:

(1) $$\begin{array}{c} \text{QTVI}=\log_{10}\left[\;\frac{\text{QTv}/{\text{QTm}}^{2}}{\text{RRv}/{\text{RRm}}^{2}}\;\right]. \end{array}$$

In this formula, QTv represents the QT interval variance, QTm is the mean QT interval, RRv is the RR interval variance, and RRm is the mean RR interval.

In order to evaluate repolarization duration we calculated average QT interval and corrected QT interval and Tp-e interval—an index considered to reflect transmural dispersion of ventricular repolarization [26]. The average absolute QT interval was normalized for RR interval variations by applying several correction formulas. In addition to Bazett's formula (QTc = QT/RR^0.5^) we used two of six regression formulas proposed by Malik et al. [10]—linear QTc = QT + *α*  × (1-RR) and parabolic QTc = QT/RR^*α*^. The rationale for choosing specifically these two formulas with two different *α* values—*α* minimal and *α* 0.2—has been presented in our previous publication [27].

### 2.4. Statistical Analysis

Unless otherwise indicated, the results are presented as means ± standard deviation (SD). Between-group comparisons of variables were carried out using Student's* t*-test. For the statistical analysis, we used Microsoft Excel version 2007 (Microsoft Corp., Redmond, WA, USA). Two-sided* P *≤ 0.05 was considered statistically significant.

## 3. Results

### 3.1. Subjects' Characteristics

The investigated population consisted of 30 male (age 40.0 ± 11.5 yr., range 21–60 yr.) subjects. The patients' mean body mass index (BMI) was 26.8 ± 4.8 kg/m^2^ and mean apnea-hypopnea index was 1.5 ± 1.4. The mean oxygen saturation values (SpO_2_) were during waking 94.8 ± 1.9%, NREM sleep 94.5 ± 1.3%, and REM sleep 94.6 ± 1.6%. Eight patients had treatment-controlled hypertension with an average blood pressure below 140/90 mmHg.

### 3.2. QT Interval Variability and Duration in Different Sleep Stages

The averaged parameters characterizing heart rate and ventricular repolarization in sleep stages are presented in Tables 1 and 2.

The *P* values of differences in characteristics of heart rate, QT interval variability, and duration are presented in Tables 3 and 4.

#### 3.2.1. NN Interval

A significantly increased mean NN (*P* < 0.05) in NREM sleep in comparison to while awake was present. NN interval means between other sleep stages were not statistically different.

#### 3.2.2. Tp-e Interval

No significant difference between Tp-e interval characteristics was demonstrated.

#### 3.2.3. QT Interval

Mean QT interval duration was significantly shorter (*P* < 0.05) while awake in comparison with NREM sleep (Table 3).

#### 3.2.4. QTVI

**Figure 1:**
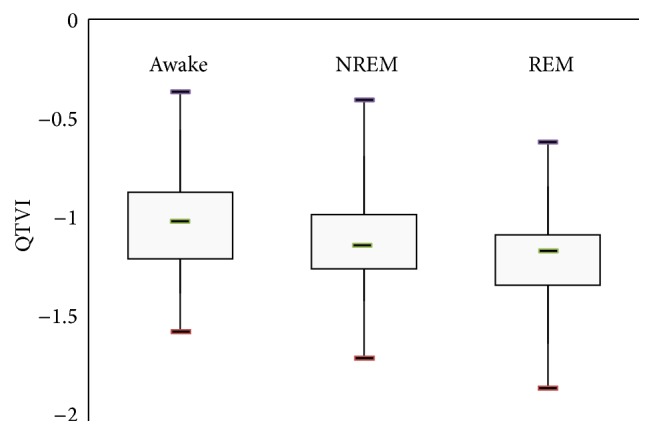
Mean values of QTVI in different sleep stages.

Mean values of QTVI while awake, in NREM sleep stage, and in REM sleep stage were −1.0 ± 0.3, −1.1 ± 0.3, and −1.2 ± 0.3, respectively. These values were similar to those of healthy controls reported by Berger et al. (−1.29 ± 0.51) [7] and similar to the weighted mean value (−1.46) for healthy volunteers outlined by Dobson et al. [28]. No statistically significant difference between stages of waking, NREM, and REM was found: *P* value of waking versus NREM is 0.22; *P* value of waking versus REM is 0.05; *P* value of NREM versus REM is 0.45 (Figure 1).

#### 3.2.5. QTc Interval

No difference was detected in mean QT interval duration corrected by Bazett's formula between sleep stages. From the results summarized in Table 4, we can deduce that the corrected QT interval values, corrected by linear (both *α* minimum and *α* 0.2) and parabolic (*α* minimum) regression models, were statistically shorter while awake in comparison with NREM sleep stage (*P* < 0.05).

## 4. Discussion

In the present study, we investigated the influence of various sleep stages on QT interval variability and duration recorded in nonapneic male subjects. Since the so-called ventricular repolarization reserve is considered to be gender-dependent [18, 29] we included male patients only. Our original approach consisted of simultaneous application of conventional ventricular repolarization duration and variability assessment formulas to polysomnographic ECG recordings at various sleep stages.

The novelty of this study lies in the previously, to the best of our knowledge, unpublished finding that in nonapneic male patients the QT interval variability assessed by QTVI is not affected by REM sleep stage; that is, the repolarization variability is not increased compared to NREM sleep and waking.

### 4.1. Impact of Various Sleep Stages on the QT Interval Variability Index

Increased temporal ventricular repolarization variability, assessed by QTVI, is convincingly proven to be associated with elevated risk for sudden cardiac death in various cardiac diseases [11, 14, 30–32] and noncardiac conditions [13]. Moreover, it is considered that the augmented QT variance, rather than a drop in heart rate variance, is responsible for increased QTVI in cardiac patients [33]. In recent years, obstructive sleep apnea-related arrhythmogenic risks have increasingly received attention by medical community; at the same time only limited data exist on QTVI as a parameter predicting ventricular arrhythmias in sleep-related breathing disorders [16, 34]. So far, our knowledge concerning the physiological effects of normal sleep on the temporal QT variability is limited. Thus, it is essential to learn more about the fluctuations of this parameter during normal sleep and take such fluctuations into account while interpreting changes in ventricular repolarization in pathological conditions.

It is well known that in alternating sleep stages autonomic cardiac control fluctuates between sympathetic and parasympathetic predominance. The data presented by Somers et al. [35] indicated that in normal subjects sympathetic activity is lower when they are in deep NREM sleep than during waking and REM sleep. Moreover, it is accepted that the sympathetic activity surges during REM sleep can exceed the levels recorded at waking.

QT interval beat-to-beat variability is influenced by multiple factors and, although the mechanisms contributing to variability are not completely understood, elevated sympathetic activity has been considered to be one of the most important among them [20, 36]. Therefore it is tempting to presume that sympathetic bursts in REM sleep have influence on ventricular repolarization temporal lability. However, the results of our study do not prove that sleep stages affect QT variability, at least in subjects without OSA. We suggest that physiological surges (signal inputs) in sympathetic activity characterizing REM sleep do not lead to clinically significant fluctuations in beat-to-beat dynamicity of ventricular repolarization. Our findings are thus indirectly consistent with the widely accepted opinion that temporal fluctuations in repolarization are aroused by elevated cardiac sympathetic activation in certain pathological conditions only [20, 36]. We conclude that, in the absence of substantial structural and electrical remodeling of myocardium, physiological elevation in sympathetic activity during REM sleep remains subthreshold for clinically significant increase of myocardial electrical instability.

### 4.2. Impact of Various Sleep Stages on QT and QTc Interval Duration

During continuous ECG recordings, QT interval duration exhibits a well-known circadian pattern reflecting waking- and sleep-induced alterations in ventricular repolarization. Previous studies focusing on the effect of sleep and its stages on the QT interval have established that both QT and QTc intervals are longer at nighttime [16, 18, 37, 38]. In the earlier studies, evaluations were made from 24-hour ECG recordings, where sleep was assumed and changes in sleep stages were not taken into consideration. Gillis et al. [39] studied 8 nonapneic patients with frequent ventricular ectopy at polygraphic monitoring and noted certain prolongation of QT and QTc in NREM as well as REM sleep compared to waking, with no difference between REM and NREM sleep. Unfortunately factors such as small sample size and coexisting cardiac disease limit the interpretation of the data. Lanfranchi et al. [18] investigated healthy individuals free of any sleep disorder using polysomnography and found that the heart rate-corrected QT interval in men remained stable from waking through all sleep stages.

In our study the absolute mean values of QT interval during NREM sleep increased significantly when compared to waking, which can be expected from the relative increase in vagal tone during NREM sleep stage. The differences between waking and REM sleep, as well as between REM and NREM sleep did not reach statistical significance.

Various rate-corrected QT interval formulas grant possibility for obtaining QT values independent of the corresponding RR interval values. The discrepancy among the studies suggests that the formulas have been influenced by the individual differences in the QT/RR relation. In order to determine the dynamics of QT interval, analyzing just RR interval preceding QT interval may be inadequate. The independence of QT interval from previous RR interval can be tested by computing the correlation coefficients. That is why linear and parabolic correction formulas with two different *α* values were used in our study.

We found that the mean QTc intervals, calculated by different formulas, slightly lengthened in NREM and REM sleep. The difference did not reach statistical significance when Bazett's formula was applied. While using linear and parabolic correction formulas the difference of QTc was significant between waking and NREM sleep in some occasions (Table 4), which according to our opinion is not clinically relevant. On the contrary, the absence of difference of the mean QTc interval values between NREM and REM stages, calculated by all formulas, seems to confirm our conclusion that sympathetic surges during REM stage do not affect repolarization phase.

Finally, some researchers have suggested that prolongation of T-wave apex to T-wave end (Tp-e) interval reflects transmural heterogeneity of ventricular repolarization and can therefore be used as a surrogate marker for risk of elevated reentrant ventricular arrhythmias [26]. Increased Tp-e interval has been reported to be associated with potentially life-threatening ventricular arrhythmias and SCD [40], as well as in patients with moderate-to-severe OSA [17]. That is why we considered it worthwhile to assess the behaviour of this parameter during REM sleep. Similar to the other ventricular repolarization parameters, the mean Tp-e interval was not prolonged at REM sleep as compared to waking or NREM sleep (Table 3).

### 4.3. Limitations of the Study

We attempted to achieve maximal homogeneity in the physical status of the patients investigated. Nevertheless, a certain number of patients with mild hypertension, although drug-controlled and without clinically detectable left ventricular hypertrophy, were included. Hypertension has been proved to be associated with sympathovagal imbalance [41].

The same applies to obesity. Although we excluded patients with BMI ≥ 35 kg/m^2^, our patients' mean body mass index 26.8 ± 4.8 kg/m^2^ indicates that several patients were clinically overweight. Obesity has been shown to increase QTVI [42]. However, the normal values of QTVI as well as QTc obtained by 5 different regression formulas during REM sleep demonstrate the limited effect of these factors on ventricular repolarization in our study.

It has been reported that REM sleep at the end of the night is characterized by more intensive sympathetic modulation compared to REM sleep that occurs during the first part of the night. From a clinical point of view, this fact can be a potential link between increased sympathetic drive, REM sleep, and the incidence of cardiovascular events in the early morning [43]. We did our best to perform measurements during the REM episodes in the morning hours; however, a few midnight REM episodes were included into data processing. We presume that the limited number of these episodes do not have notable effect on the results.

## 5. Conclusions

Our results reveal that, in male patients without OSA, different sleep stages do not affect QTVI values. In the absence of notable structural and electrical remodeling of myocardium, physiological elevation in sympathetic activity during REM sleep remains subthreshold concerning clinically significant increase of myocardial electrical instability. Elevated QTVI values found in patients with OSA cannot be interpreted as physiological sympathetic impact during REM sleep and should be considered as a risk factor for potentially life-threatening ventricular arrhythmias. The absence of difference of mean QTc interval values between NREM and REM sleep stages applying all formulas seems to confirm our conclusion that sympathetic surges during REM stage do not induce repolarization variability. Significant difference in QTc between waking and NREM sleep obtained in our study by certain heart rate correction formulas seems to have no clinical importance as the mean values of the parameter remained in physiological range. The presence and the extent of polysomnographically recorded QTVI and QTc changes in OSA patients require further thorough investigations and require the gender-dependence of these changes.

## Figures and Tables

**Table 1:** Characteristics of heart rate, QT interval duration, and variability in sleep stages.

Variables	Awake	NREM	REM
NN (ms)	962.4 ± 126.3	1032.0 ± 117.5	973.6 ± 109.1
QT mean (ms)	374.6 ± 24.6	389.5 ± 23.1	382.1 ± 23.8
Tp-e (ms)	72.4 ± 7.2	75.9 ± 6.6	74.3 ± 6.4
QTVI	−1.0 ± 0.3	−1.1 ± 0.3	−1.2 ± 0.3

Data are expressed as means ± SD; NREM: nonrapid eye movement sleep stage; REM: rapid eye movement sleep stage; NN: Normal-to-Normal interval; QT: QT interval; Tp-e: T-wave peak to T-wave end interval; QTVI: QT variability index.

**Table 2:** Characteristics of rate corrected QT intervals in sleep stages.

Variables	Awake	NREM	REM
QTc Bazett mean (ms)	383.7 ± 18.4	384.7 ± 19.9	388.8 ± 19.9
QTc Lin *α* min mean (ms)	376.2 ± 22.1	389.7 ± 21.7	384.2 ± 21.6
QTc Par *α* min mean (ms)	376.0 ± 22.3	389.6 ± 21.7	384.1 ± 21.7
QTc Lin *α* 0.2 mean (ms)	374.5 ± 24.6	389.3 ± 23.0	382.0 ± 23.8
QTc Par *α* 0.2 mean (ms)	377.9 ± 18.6	387.3 ± 18.9	384.5 ± 19.8

Data are expressed as means ± SD; NREM: nonrapid eye movement sleep stage; REM: rapid eye movement sleep stage; QTc Bazett: rate corrected QT interval based on Bazett's formula; QTc Lin *α* min: rate corrected QT interval based on linear regression formula *α* minimum; QTc Par *α* min: rate corrected QT interval based on parabolic regression formula *α* minimum; QTc Lin *α* 0.2: rate corrected QT interval based on linear regression formula *α* 0.2; QTc Par *α* 0.2: rate corrected QT interval based on parabolic regression formula *α* 0.2.

**Table 3:** The *P* values of heart rate, QT interval variability, and duration between sleep stages.

Variables	Awake/NREM	Awake/REM	NREM/REM
	*P* value		
NN	0.03	0.71	0.05
QT mean	0.02	0.23	0.23
Tp-e	0.05	0.28	0.33
QTVI	0.22	0.05	0.45

Significance of differences between sleep stages: *P* < 0.05; REM: nonrapid eye movement sleep stage; REM: rapid eye movement sleep stage; NN: Normal-to-Normal interval; QT: QT interval; Tp-e: T-wave peak to T-wave end interval; QTVI: QT variability index.

**Table 4:** The *P* values of rate corrected QT intervals between sleep stages.

Variables	Awake/NREM	Awake/REM	NREM/REM
	*P* value		
QTc Bazett mean	0.81	0.30	0.44
QTc Lin *α* min mean	0.02	0.16	0.34
QTc Par *α* min mean	0.02	0.16	0.33
QTc Lin *α* 0.2 mean	0.02	0.23	0.23
QTc Par *α* 0.2 mean	0.06	0.19	0.58

Significance of differences between sleep stages: *P* < 0.05; REM: nonrapid eye movement sleep stage; REM: rapid eye movement sleep stage; QTc Bazett: rate corrected QT interval based on Bazett's formula; QTc Lin *α* min: rate corrected QT interval based on linear regression formula *α* minimum; QTc Par *α* min: rate corrected QT interval based on parabolic regression formula *α* minimum; QTc Lin *α* 0.2: rate corrected QT interval based on linear regression formula *α* 0.2; QTc Par *α* 0.2: rate corrected QT interval based on parabolic regression formula *α* 0.2.
